# *Cissus quadrangularis* L extract-loaded tricalcium phosphate reinforced natural polymer composite for guided bone regeneration

**DOI:** 10.1007/s10856-023-06739-x

**Published:** 2023-07-19

**Authors:** Lele Liao, Weihong Zhu, Cheng Tao, Ding Li, Minzhi Mao

**Affiliations:** Department of Orthopedics, The Second Xiangya Hospital, Central South University, Changsha, Hunan 410011 China

## Abstract

Natural medicines plants are significant considerable attention as potential therapeutic agents for bone tissue engineering. *Cissus quadrangularis* L (CQ). is a potent therapeutic plant known for its own osteogenic properties. In this research work, a phytoconstituents-filled composite was produced by incorporating CQ extract with gelatin (Gel) and pectin (Pec) polymers collective through β- tricalcium phosphate (β-TCP) bioceramic via a green template method. The effect of CQ-filled composite morphology and chemical structural properties, in vitro cytotoxicity, cell proliferation, and differentiation was investigated. FTIR spectroscopic results indicated the prepared materials’ structural confirmation. The CQ extract was the alcoholic -OH merge with the hydroxyl and -NH groups in the range of 3000 cm^−1^ to 3500 cm^−1^. Scanning electron microscopy images showed that the β-TCP ceramic was perfectly embedded in Gel-Pec polymeric matrix, which is important for bone regeneration. In vitro cell culture results indicated that β-TCP/Gel-Pec/CQ composite provided 92.0% of a favorable substrate for mesenchymal stem cell viability. The gene expression and RT-PCR studies represent the materials with good osteogenic expression, especially the β-TCP/Gel-Pec/CQ composite is observed at 168.0% and 188.0% for RUNx2 and OCN, respectively. The result of the physicochemical characterizations and cell viability studies suggest that CQ-loaded β-TCP/Gel-Pec composite can serve as a potential biomaterial for bone tissue repair and regeneration.

**Graphical Abstract:**

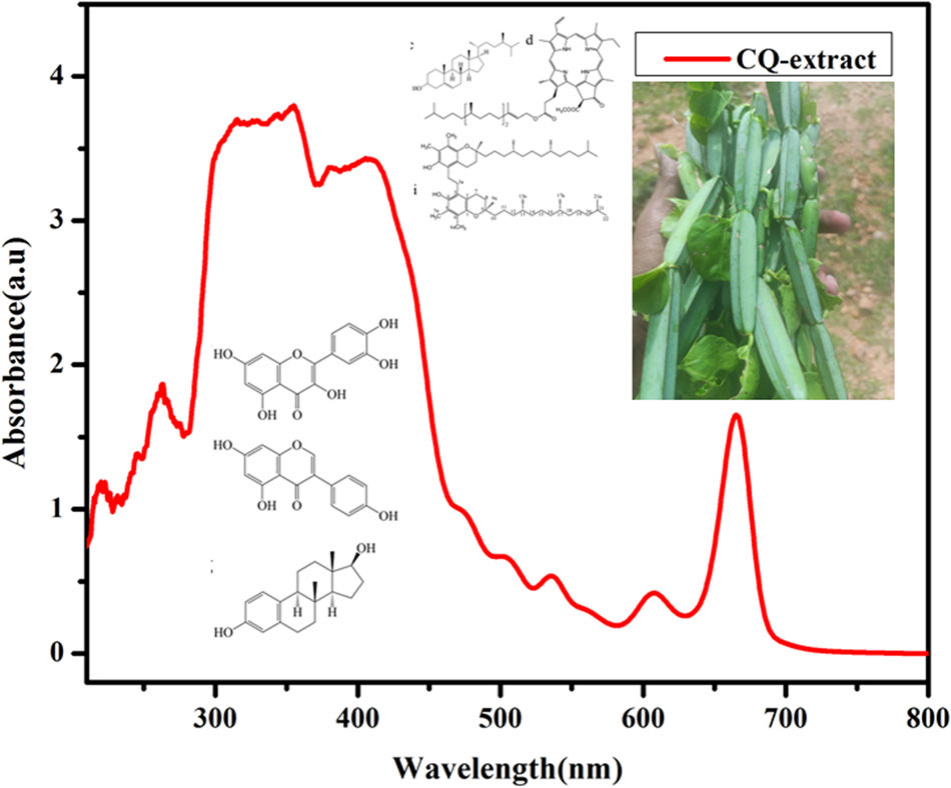

## Introduction

The specific advantages like the absence of toxicity, morbidity, and their brilliant biocompatible nature, bone biomaterials have attracted more consideration over existing graft treatments in recent years. It aims to recover, preserve and encourage the functions of injured organs from a group of regenerative cells proficient in self-healing and differentiation in other cell types [1–3]. Subsequently, developing a scaffold with the finest architecture, high porosity, and appropriate pore size is the most vital factor for bone tissue engineering [4]. Numerous types of polymers, including natural sources like gelatin (Gel) [5], collagen (COL) [6], pectin (Pec) [7], and synthetic polymeric materials like oxidized alginate [8], hyaluronic acid (Hya) [9], poly(DL-lactide-co-glycolide) (PLGA) and poly(L-lactic acid) (PLLA) [10] have been examined, for the persistence of applications including tissue regenerations and drug delivery, as of their excellent medicinal activities, their biocompatibility, their biodegradability properties, and their cost-effective nature.

Among all the above polymers, natural polymers such as Gel and Pec are highly interested polymers in bone tissue regenerating applications due to their better biocompatible, non-toxic, and cellular adhesion ability. The Gel is a hydrolysis product of collagen, a water-soluble protein, and collagen is naturally present in the natural bone as an organic component [11]. Besides, the comfort of the chemical alteration of Gel and its marketable availability has driven the widespread claim of biomaterial in tissue regeneration along with bioactive compound releases [12]. However, gelatin has better cell-differentiating, proliferating, and migration abilities. Polysaccharides like pectin can increase their biocompatible and cell-differentiating behavior [13]. The scaffold can also be suitable for tissue engineering applications. Many heteropolysaccharides from various components of plant cell walls are composed and create the natural carbohydrate pectin [14, 15]. Pectin has expanded responsiveness due to numerous distinct properties, such as extraordinary water content and the ability of homogenous immobilization of cells, proteins, therapeutics, and growth factors.

Furthermore, pectin can act as an ideal carrier for drug delivery applications because of its capability to form a gel in the presence of divalent cations [16, 17]. In 2015, Amirian et al. synthesized the Gel-Pec polymeric composite for loading VEGF and BMP-2 with calcium phosphate ceramic loading bone growth factors [18]. The Gel-Pec-BCP scaffold can act as a biopolymer composite scaffold with VEGF and BMP-2 growth factors for tissue regeneration applications [18]. In 2019, Mehrali et al. prepared the methacrylate Pectin (PEMA) hydrogel, which was then incorporated with Gel polymer to make the hydrogel more cell friendly [19]. They found that the PEMA-Gel hydrogel showed an astonishing collection of the highly wanted properties in this bone regeneration field. The hydrogel successfully converted hBMSCs into bone cells, indicating the cell-differentiating ability of the PEMA-Gel hydrogel [19].

Then again, the accelerated bone redevelopment was reported in the existence of ceramics comprising calcium phosphate (CP) and hydroxyapatite (HAp), which makes them appropriate for the scaffolds for bone tissue engineering [20, 21]. Moreover, the brittle nature of ceramics can be overcome with polymers for effective bone healing. The osteoconductive nature of the Gel-Pec polymeric scaffold was increased by adding biphasic calcium phosphate (BCP) for effective bone regeneration was achieved by Amirian et al. in 2015 [18]. Moreover, the addition of BCP nanoparticles on the Gel-Pec scaffold encouraged faster tissue regeneration in vivo when implanted in the rabbit femur [12]. In this case, we have loaded the osteoinductive tricalcium phosphate (TCP) ceramic on the Gel-Pec polymeric scaffold for guided bone regeneration. With its adjustable pore size, porosity, and roughness, TCP demonstrates significant benefits for regenerating and repairing bone tissues. Additionally, the mechanical performance of TCP can be enhanced by the addition of various materials to modify its hardness and capacity to deteriorate. Due to its natural bioactivity, the porous structure of TCP can operate as bioactive compound carriers to transfer pro-osteogenic growth hormones with favorable releasing kinetics and less adverse effects. The distribution of therapeutic ionic dissolution from TCP is a more acceptable breakdown rate than other materials [22].

In addition, the utilization of natural products in bone biomaterials is highly interesting because of the presence of various nutrients, vitamins, and phytochemicals in these natural products used as drug materials for many uncontrolled situations since the ancient period [23]. Natural compounds such as garlic, ginger [24], curcumin [25], kaempferol, quercetin, peperomia pellucid (PP), aloe vera (AV) [26], etc., were described before for their bone constructive ability. *Cissus quadrangularis* (CQ) is a medicinal plant since the ancient period with osteogenesis activity for bone curative nature due to its prospective of Phyto-estrogenic steroids, bone estrogenic receptors, carotene, ascorbic acid, and calcium minerals [27]. Previously, CQ was employed as the orthopedic setters of the composites such as alginate/O-carboxymethyl chitosan (O-CMC) and chitosan/Na-carboxymethyl cellulose for bone tissue engineering [28, 29]. Prabakaran et al. recently fabricated a nanofibrous composite containing minerals substituting HAP/PEG/CQ materials [30]. They found that adding CQ has encouraged osteoblasts proliferation and differentiation on bone-like MG-63 cells without producing toxic effects [30]. The novelty of the current research is the attempt at natural medicine filled β-TCP/Gel-Pec/CQ composite. *Cissus quadrangularis* L (CQ). is a potent therapeutic plant known for its own osteogenic properties, and the porous structure of TCP with reinforced pectin and gelatin composite can operate as bioactive compounds carriers to transfer pro-osteogenic growth hormones with favorable releasing kinetics and with less adverse effects. Based on this investigation, we have achieved the TCP/Gel-Pec/CQ composite for guided bone regeneration.

## Experimental section

### Materials

The materials required to prepare the composite contained Strontium nitrate, Calcium nitrate tetrahydrate, Diammonium hydrogen phosphate, Ammonia solution, Gelatin, Pectin, and Ethanol were purchased from Sigma–Aldrich, China. Sodium chloride, Calcium chloride, Potassium hydrogen phosphate, Sodium bicarbonate, Potassium chloride, Magnesium chloride, 1 M Hydrogen chloride, and Tris buffer were purchased from Sigma–Aldrich for the preparation of the SBF solution.

### Preparation of β-tricalcium phosphate (β-TCP)

Initially, 100 mL of 0.2 mol (NH_4_)_2_HPO_4_ solution and 100 mL of 0.3 mol of Ca(NO_3_)_2_ .4H_2_O solution were prepared by dissolving the corresponding salts in water. Then, by a burette, Ca(NO_3_)_2_.4H_2_O solution was added to the (NH_4_)2HPO_4_ solution in a drop-wise manner. The co-precipitation was achieved at a pH value of 9.0 which was achieved by using an ammonia solution. The stirring was continued for 12 h until a white precipitate was obtained. The obtained residue was washed with DD (double distilled) water and 95% ethanol, and the white precipitate was subsequently filtered. The as-obtained filter cake was desiccated for 24 h at 80 °C. The desiccated powder was crushed by mortar and pestle. Then the obtained β-TCP powder was calcinated at 900 °C for 2 h in a muffle furnace and confirmed by XRD analysis.

### Preparation of β-TCP/Gel-Pec composite

150 mg of pectin was soluble in 5 mL of an aqueous solution. Then 30 mg of EDC (N-(3-dimethylaminopropyl)) N-ethylcarbodimide hydrochloride and 12 mg NHS (N-hydroxysuccinimide) were mixed in the pectin solution and stirred for 1 h. Then, the Gelatin solution (150 mg of gelatin in 5 mL water) was added to the pectin solution and mixed for 3 h. After 3 h of the stirring period, then 150 mg of β-TCP distributed in 10 ml of water was added to the Gel-Pec solution. The reaction was carried out for 24 h under a magnetic stirrer, and then the solution was collected in a watch glass and dried in a Hot air oven. After the composite was grained, the β-TCP/GEL-PEC composite was collected and stored on vials.

### Preparation of CQ extract

The collected CQ stem was washed thoroughly with DD water, dried in the shade, cut into small pieces, and mechanically powdered. 10 g of the very small particles CQ stem was soaked in 50 mL of absolute ethanol, stirred for 12 h, and filtered. The extraction of CQ stem was separated and weighed; 1.0 g of extract was obtained. Further, UV-Visible spectroscopy analysis was carried out to know the presence of phytoconstituents in the CQ extract.

### Preparation of β-TCP/Gel-Pec composite in the template method

(NH_4_)_2_HPO_4_ solution and Ca(NO_3_)_2_ solution were used as the raw materials for the presence of Ca, PO_4_, Gelatin, and Pectin were used as the template for the preparation of β-TCP. Firstly, 150 mg of pectin was dissolved in 5 mL of water under a magnetic stirrer and stirred well for 30 min. After 30 min, 150 mg of gelatin dissolved in 5 mL of water was added to the pectin solution and stirred for 1 h. Then, 25 mL of 0.3 mol of Ca (NO_3_)_2_ .4H_2_O solution was added, and the reaction was carried out for 2 h. After the completion of the reaction, 25 mL of 0.2 mol (NH_4_)_2_HPO_4_ solution was added slowly into the above mixture solution. The co-precipitation process was performed at a pH 9.0 using an ammonia solution. The mixing process was continued for 12 h, until a white precipitate was obtained. The reaction product was centrifuged, washed with ethanol and water, and dried in a hot air oven. The composite prepared by the two methods composites investigated their morphology by SEM technique. The β-TCP prepared by the templated method was observed to have good morphology since it was used for further composite formation.

### Preparation of CQ extract-loaded β-TCP/Gel-Pec composite (Template method)

The final composite of β-TCP/GEL-PEC/CQ from the templated methods is used by adding 0.5 g of CQ extract to the above previous solution before centrifuging it. After the extract addition, the reaction was carried out for 24 h. Then the reaction product was centrifuged and dried in a Hot air oven. After the composite was grained, the β-TCP/Gel-Pec/CQ composite was collected and stored on vials.

### Physicochemical characterizations

#### Functional group analysis using Fourier transform infrared (FTIR) spectroscopy

The FTIR spectroscopy obtained from IRTRACER-100, Shimadzu, was utilized to evaluate the functional groups present in β-TCP ceramic and all prepared composites, as well as the interactions between the components in the composites. Spectra of the prepared samples with a KBr pellet were attained in the interior of the scanning range of 4000–400 cm^−1^.

#### Phase analysis using X-Ray diffraction (XRD) analysis

The phase behavior and the crystallinity of the prepared β-TCP ceramic and the composites based on β-TCP ceramic were evaluated using an XRD instrument from Bruker ECO D8 Advance model. The scanning progression was achieved by functioning the instrument with monochromatized Cu anode at 40 kV and 25 mA scanning angle from 10° to 80° and scanning rate (2θ) of 10.

#### Morphology analysis using scanning electron microscope (SEM)

The β-TCP ceramic and the composites’ surface morphology were inspected by the SEM instrument fortified with Energy Dispersive X-ray (EDX) from VEGA3 TESCAN. All water-dispersed inspected samples were coated on the glass plate and then dried at 27 °C for SEM scanning.

#### Transmission electron microscope (TEM) analysis

The microstructure of the prepared composite was inspected with Transmission Electron Microscope (HR-TEM) instrument obtained from FEI Technai G220 S- TWIN TEM. The water-dispersed sample in the SEM experiment was also recycled to take TEM microstructure by coating the dispersed samples on the Cu grid. The average diameter of the composites was estimated by image J software.

#### Preparation of SBF Solution

300 mL of DD water was taken in 500 mL of the plastic beaker and stirred under a magnetic stirrer. 2.4 g of NaCl was weighed and added to the beaker. It was then stirred well to dissolve completely. Then, 0.11 g of NaHCO_3_, 0.07 g of KCl, 0.07 g of KHPO_4_, 0.09 g of MgCl_2_, 0.09 g of CaCl_2_, 0.022 g of Na_2_SO_4_, 1.8 g Tris buffer, and 12 mL of 1 M HCl in the water solution were added one by one after completion of dissolve in each salt. The pH of the reaction maintains a neutral (pH -7.0), adding either HCl or Tris buffer. The SBF solution was stored in a refrigerator [31].

### Biological studies

#### In vitro cell culture

The human bone marrow mesenchymal stem cells (hBMSCs) were bought from the American type culture collection (ATCC PCS-500–012) and were cultured in the 24-well tissue culture plates in the presence of Dulbecco’s Modified Eagle Medium (DMEM, GIBCO), minimal essential media (Hi-Media Laboratories) and the 10% Fetal Bovine Serum (FBS). The antibiotics streptomycin (100 U mL^−1^) and Penicillin (100 U mL^−1^) were given for 48 h to avoid bacterial infections. Afterward, the normal media was replaced by renewed growth media. Further, the growth condition was maintained in the humidified atmosphere containing 95% air and 5% CO_2_ at 37 °C [32].

#### hBMSCs attachment and proliferation

Before seeding the cells, all testing samples were immersed in DMEM/F12 (50:50 ratio) with minimal essential media and 10% FBS for 150 min. Before this immersion, all samples were sterilized with alcohol (75%) and washed thrice with PBS solution. The cells without any treatment are set as control. After detaching at different periods, i.e., 1, 3, 7, and 14 days, cells were seeded into new 96-well cell plates with the density of 2 × 10^4^, and 1 × 10^4^ cells per well to evaluate the cell attachment and proliferation, respectively, and maintained 24 h. Now, in serum-free medium, it was added the 2 mL of MTT (3-(4,5-dimethyl-2-thiazolyl)-2,5-diphenyl-2H-tetrazolium bromide) solution to each sample, and it developed under a humidified atmosphere containing 5% CO_2_ for 4 h at 37 °C. Now, the formed formazan crystals were dissolved by adding 10% DMSO solution. The composites’ cell viability was evaluated by calculating the optical density (OD) values at 570 nm on the spectrophotometric microplate with the below equation [33]. Triplicates were averaged in this experiment.

$$\% \,{{{\mathrm{of}}\, {\mathrm{cell}}\, {\mathrm{viability}}}} = \left[ {{{\mathrm{A}}}} \right]_{{{{\mathrm{test}}}}}/\left[ {{{\mathrm{A}}}} \right]_{{{{\mathrm{control}}}}}\,{{{\mathrm{X}}}}100$$

#### Osteogenic differentiation analysis

The osteogenic differentiation determination was carried out in the osteogenic marker genes Runt-related transcription factor (RUNx), Osteocalcin (OCN), and Vascular Endothelial Growth Factor (VEGF); the mRNA levels were observed by utilizing Real-Time Polymerase Chain Reaction (RT-PCR). The specimens were cleaned with PBS and suspended in 1 mL of cold TRIzol Reagent (Life Technologies Co.) after 1, 3, 7, and 14 d culture. For the total RNA extraction of every sample, the standard TRIzol procedure was followed, and after extract, they were resuspended in the medium of 50 μL of RNase-free water. Then, cDNA was generated by utilizing the procedure of transcriptase reaction mix (SuperScript III First-Strand Synthesis System, Life Technologies). Then, the generated cDNA was stored at −20 °C. Quantitative PCR analysis was performed by utilizing a power SYBR green RT-PCR kit (Life Technologies) procedure (*n* = 3). An endogenous housekeeping gene, Glyceraldehyde-3-phosphate dehydrogenase (GAPDH), was used to define the other gene-relative transcripts [34].

#### Western blot analysis

Bovine serum albumin (BSA), a protein standard, was used to measure cell lysates’ protein concentration using the Bio-Rad detergent-compatible (Dc) microprotein assay. Depending on the protein concentration, cell lysates were heated for 5 min at 110°C after being diluted in RIPA buffer to the gel-loading concentration of proteins (3 mg/mL). Protein samples were separated by electrophoresis on 10% SDS gel (Bio-Rad, Hercules, CA, United States). The isolated proteins were transferred to the PVDF membrane Immunoblot (Bio-Rad, United States) by the wet transfer technique. The membrane was then treated with mouse monoclonal IgG primary antibodies (Osteocalcin, Cat # SC-73464; Runx2, Cat #SAB1412665 from Sigma–Aldrich, India) at 4 °C overnight after being blocked for 1 h at room temperature with 5% non-fat milk in PBS. Following the incubation, the membrane was washed three times with TBS containing 0.1% Tween-20 (5–10 min each time). Anti-mouse horseradish peroxidase-conjugated secondary antibody (goat anti-mouse IgG antibody, Cat # AP308, Sigma–Aldrich, India) was used to identify the immunoblots. We used GAPDH as a loading control. Thermo Fisher Scientific’s Super Signal TM West Pico PLUS Chemiluminescent Substrate was used with Bio-Chemi Rad’s Doc Imaging system to capture band intensity, and Image J was used to measure band intensity.

### Statistical analysis

One-way ANOVA within origin pro 8.5 was employed to achieve the statistical analysis of the investigational groups. The significance level was set as *p* < 0.05.

## Results

**Fig. 1:**
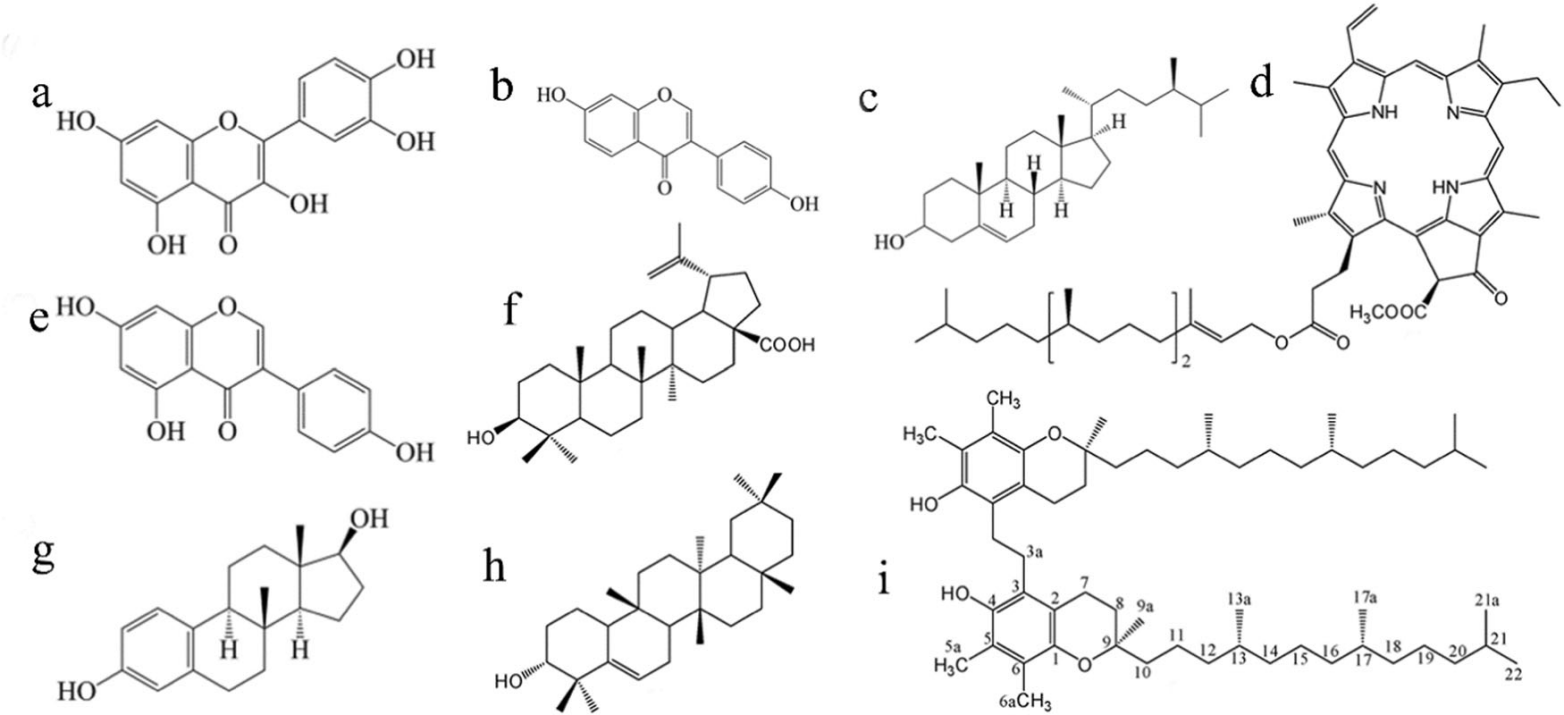
Chemical structures compounds in the *Cissus quadrangularis* Linn extract **a** quercetin, **b** diadzein, **c** β-sitosterol, **d** Pheophytin-a, **e** genistein, **f** betulinic acid, **g** estradiol, **h** beta amyrin, **i** 1,2-bis-(5-γ-tocopheryl)ethane [50]

**Fig. 2:**
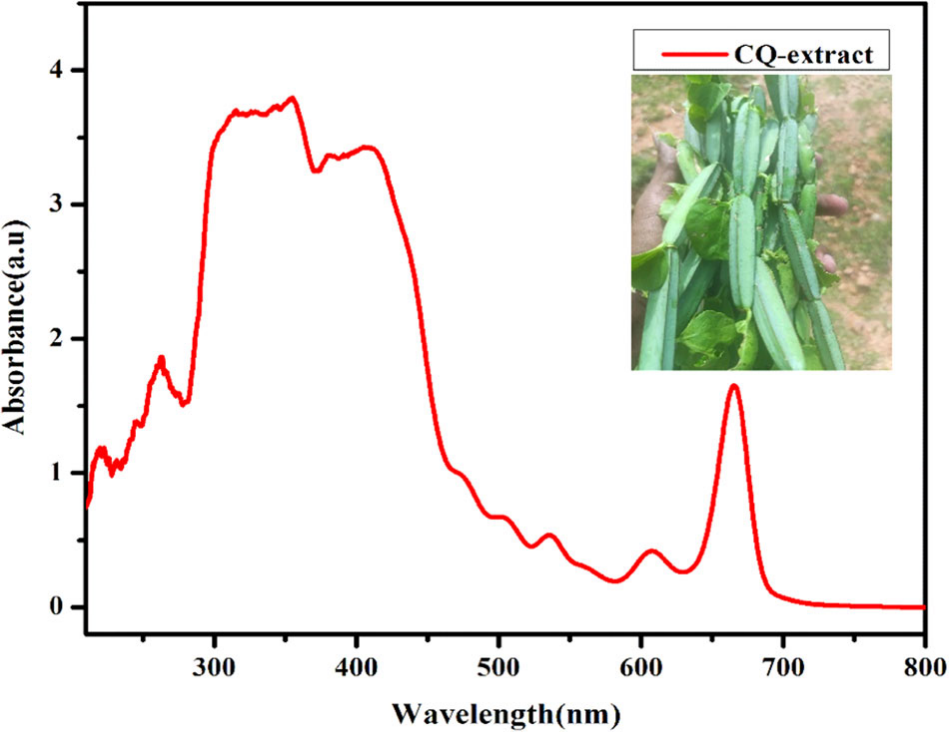
A UV-Visible spectrum of methanolic extract of *Cissus quadrangularis* L

*Cissus quadrangularis* L plant is a rich amount of anti-oxidants and other bioactive compounds. Only 5–8% of phytoconstituents are present in the leaf parts, and the major components are in the stems. Researchers are focused on the stem part for the natural compound extractions; some of the natural compounds in the CQ are given in Fig. 1. CQ powder and extract form has been applied for the last few decades to enhance bone and other tissue regenerations [35]. In this investigation, the CQ extract by the methanol was used for load in the polymer ceramic composite for human bone marrow mesenchymal stem cells (hBMSCs) differentiation. The methanol extract of the CQ was determined as the chromophore by the UV-Visible Spectroscopic analysis (UV1800, Shimazho, Japan) (Fig. 2). The UV-Visible absorption of methanol of *Cissus quadrangularis* showed absorption maxima at 667, 607, 584, 546, 500, 472, 402, 382, 357, 264, 251, and 234 nm representing several natural compounds present in the methanol extract of the *Cissus quadrangularis*. Similarly, the previous researcher reported the compounds such as quercetin, daidzein, β-sitosterol, Pheophytin-a; genistein, betulinic acid; estradiol, beta amyrin, and 1,2-bis-(5-γ-tocopheryl)ethane by the spectroscopical observation of the *Cissus quadrangularis* [36, 37].

### FTIR analysis

**Fig. 3:**
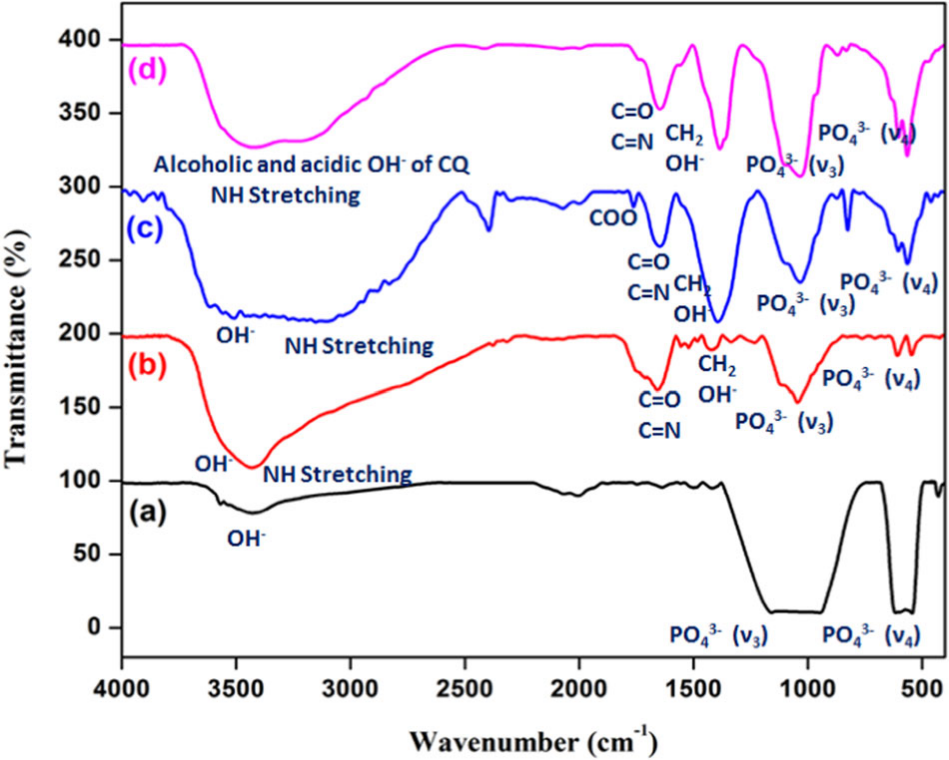
FTIR spectra of **a** β-TCP, **b** β-TCP prepared in template method, **c** β-TCP/Gel-Pec composite, and **d** β-TCP/Gel-Pec/CQ composite

FTIR is used to investigate the structural changes in the prepared materials. The results in Fig. 3 indicate the formation of the desired composites, meaning the presence of β-TCP in all the composites. All the prepared composites (Fig. 3a–d) show phosphate functional bands. β-TCP ceramic has been formed with phosphate peaks at 1417 (ν_3_) cm^−1^, as well as at 430 and 545 (ν_4_) cm^−1,^ which are characteristics of the β-TCP (Fig. 3a) [38]. Similarly, the phosphate was denoted in the formation of β-TCP in the template method, and it shows the gelatin bands at 3506 cm^−1^, which corresponds to the presence of the primary amide bond’s NH stretching (Fig. 3b). The C = O in gelatin shows an absorption peak at 1645 cm^−^^1^. The –OH stretching peak of pectin was combined with NH stretching of gelatin and was observed at 3400 cm^−1^. The peak at 1762 cm^−1^ indicated C = O stretching vibrations due to the presence of a COOCH_3_ group of pectin molecules. These bands suggest the attraction between the β-TCP ceramic and Gel-Pec molecules [18]. In this template method, the phosphate group of β-TCP changed its wavenumber due to the interaction with polymeric gelatin and Pectin (Fig. 3b). Figure 3c shows the formation of β-TCP/Gel-Pec composite, which indicates the absorption bands of all β-TCP, Gel, and Pec like Fig. 3a and Fig. 3b with the changed wavenumber of the peaks. Figure 3d represents the CQ extract-loaded β-TCP/Gel-Pec/CQ composite in which the acidic and alcoholic –OH of CQ extract was overlapped with NH stretching of Gel was observed [39]. This indicates that the CQ extract overlapped with gelatin, pectin polymers, and β-TCP ceramic to form the β-TCP/Gel-Pec/CQ composite.

### XRD analysis

**Fig. 4:**
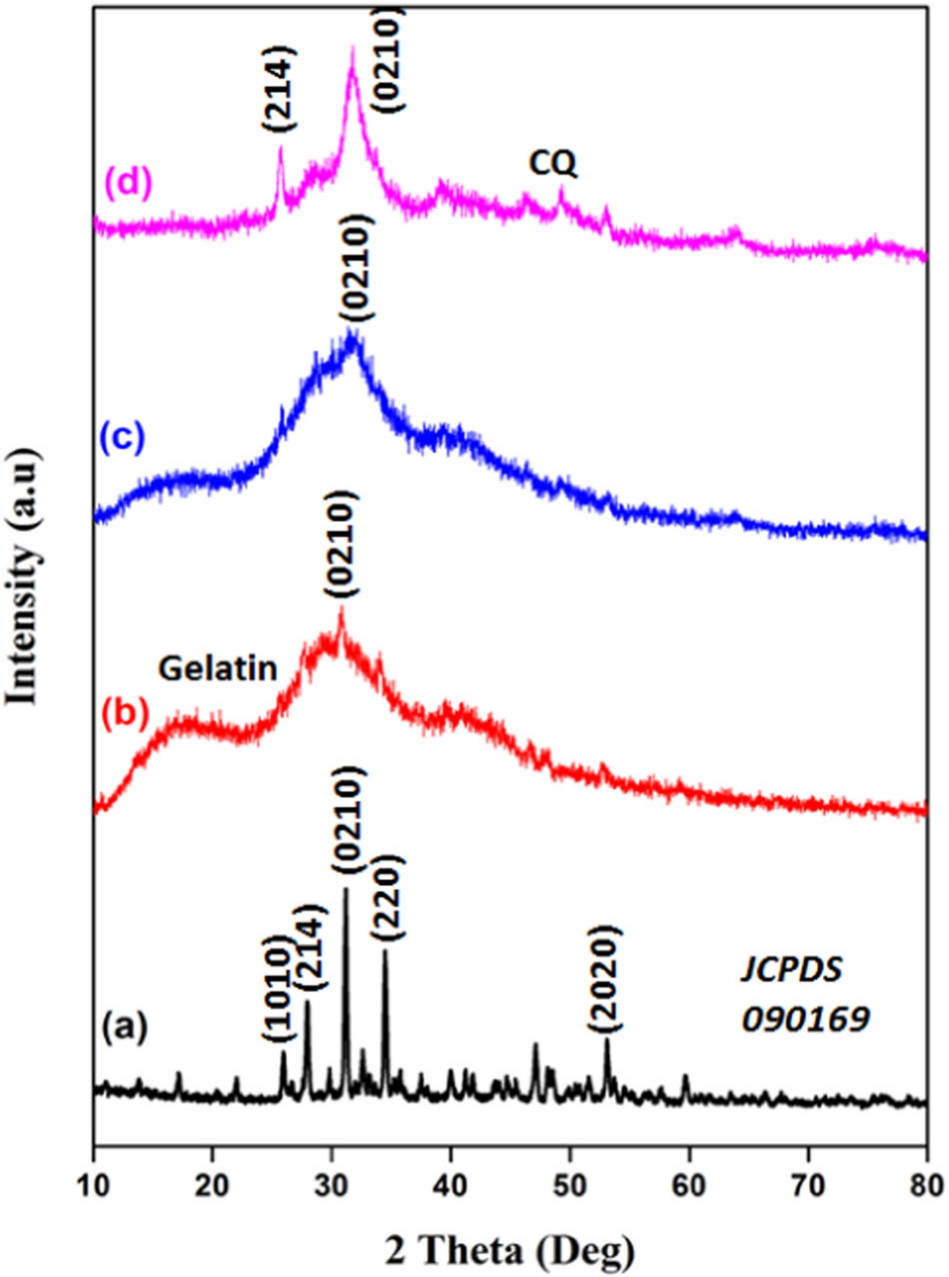
XRD spectra of **a** β-TCP, **b** β-TCP prepared in template method, **c** β-TCP/Gel-Pec composite, and **d** β-TCP/Gel-Pec/CQ composite

The XRD analysis confirms the prepared composite’s phase characteristics, which are presented in Fig. 4. The β-TCP ceramic has been formed with well crystalline nature, which can be confirmed by the appearance of sharp crystalline peaks at the 2 theta values of 25.9°, 27.9°, 31.2°, 34.5°, and 53.1° corresponding to the planes (1010), (214), (0210), (220) and (2020) respectively (Fig. 4a). This data has been well matched with previous JCPDS 090169 file [38]. The formation of β-TCP ceramic in the template method shows the broad diffraction peaks in Fig. 2b, which are the distinctive XRD pattern, representative of gelatin at the 2 theta value of 21.3° [18]. But here, the main crystalline peak of β-TCP ceramic has appeared at 30.8°, indicating the presence of β-TCP ceramic in the template. Moreover, the β-TCP/Gel-Pec composite has been formed with amorphous nature due to the fact of gelatin and pectin polymers with the retention of the main crystalline peak of β-TCP ceramic at 31.2°. The β-TCP/Gel-Pec/CQ composite has been formed with decreased crystallinity of pure β-TCP ceramic. The new CQ pattern has been formed at a 2θ value of 49.3° with the semi-crystalline β-TCP plane at 25.7° and 31.4° [30].

### Morphological analysis

**Fig. 5:**
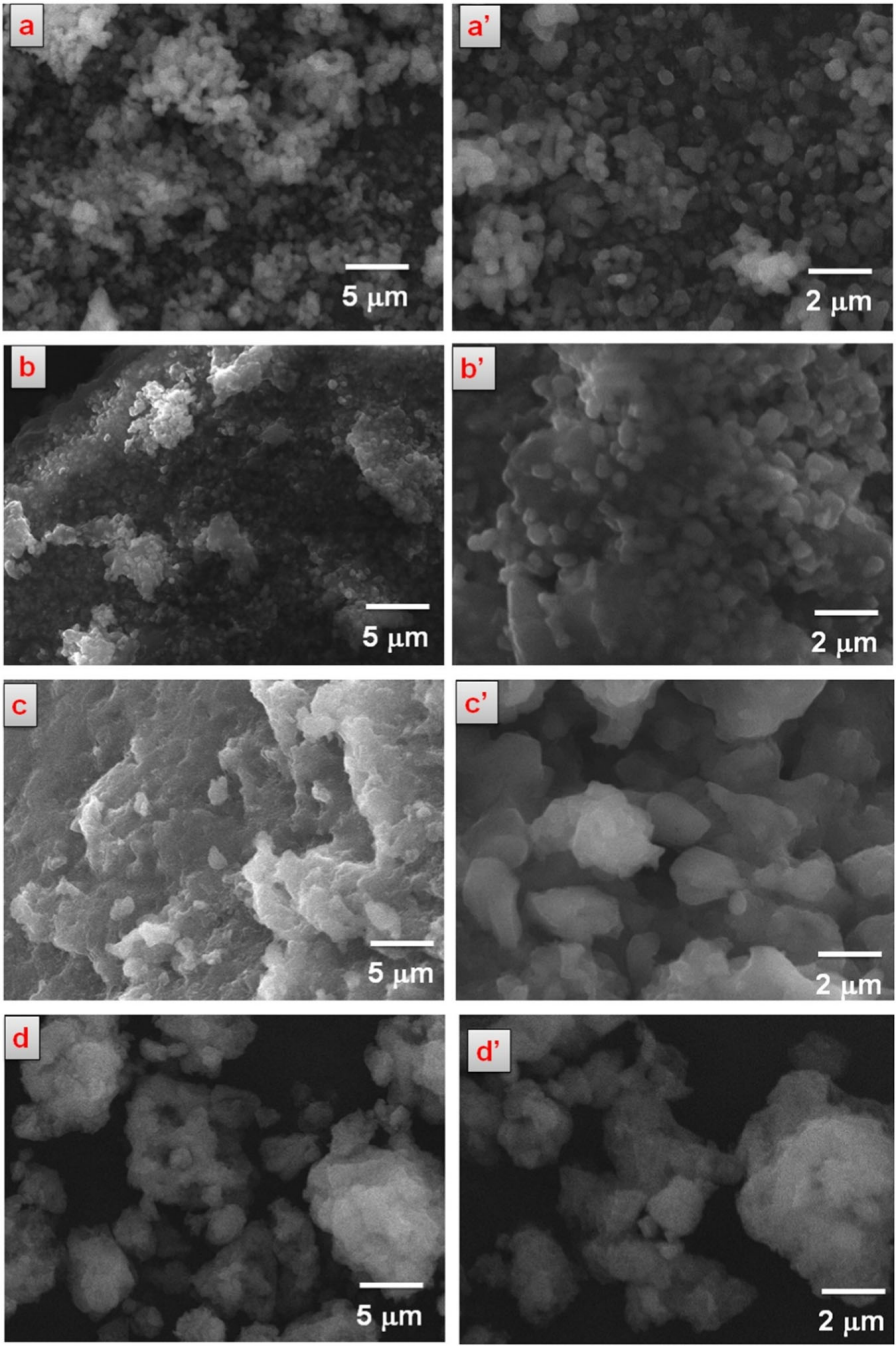
SEM morphology of Lower magnified **a** β-TCP, **b** β-TCP prepared in template method, **c** β-TCP/Gel-Pec composite, and **d** β-TCP/Gel-Pec/CQ composite. (**a’–d’**) are corresponds to their (**a–d**) components with higher magnification

Figure 5 reveals the prepared components’ low and high magnification morphology of SEM micrographs. Lower magnification of the SEM morphologies of the prepared compounds (Fig. 5a–d) reveals that the β-TCP ceramic has appeared as a particle-like morphology in all the composites and its free form. Higher magnification of the surface of the ceramics β-TCP (Fig. 5a’) and β-TCP prepared in the template method (Fig. 5b’) indicates the spherical-like morphology of the β-TCP ceramic. But after the addition of polymers such as Gel and Pec, β-TCP ceramic’s spherical-like morphology embedded in the polymeric matrix has been slightly distorted into particle-like morphology evidenced by Fig. 5c’, d’. These changes in the morphology of the β-TCP ceramic could be evidenced by the interaction of the Gel-Pec polymers with β-TCP ceramic. In addition, lower magnification of SEM images Fig. 5b, c shows that β-TCP particles were appropriately embedded in the Gel-Pec polymeric matrix in template-based synthesis and composite matrix, respectively. The addition of CQ extract does not hugely affect the morphology of the β-TCP/Gel-Pec composite.

**Fig. 6:**
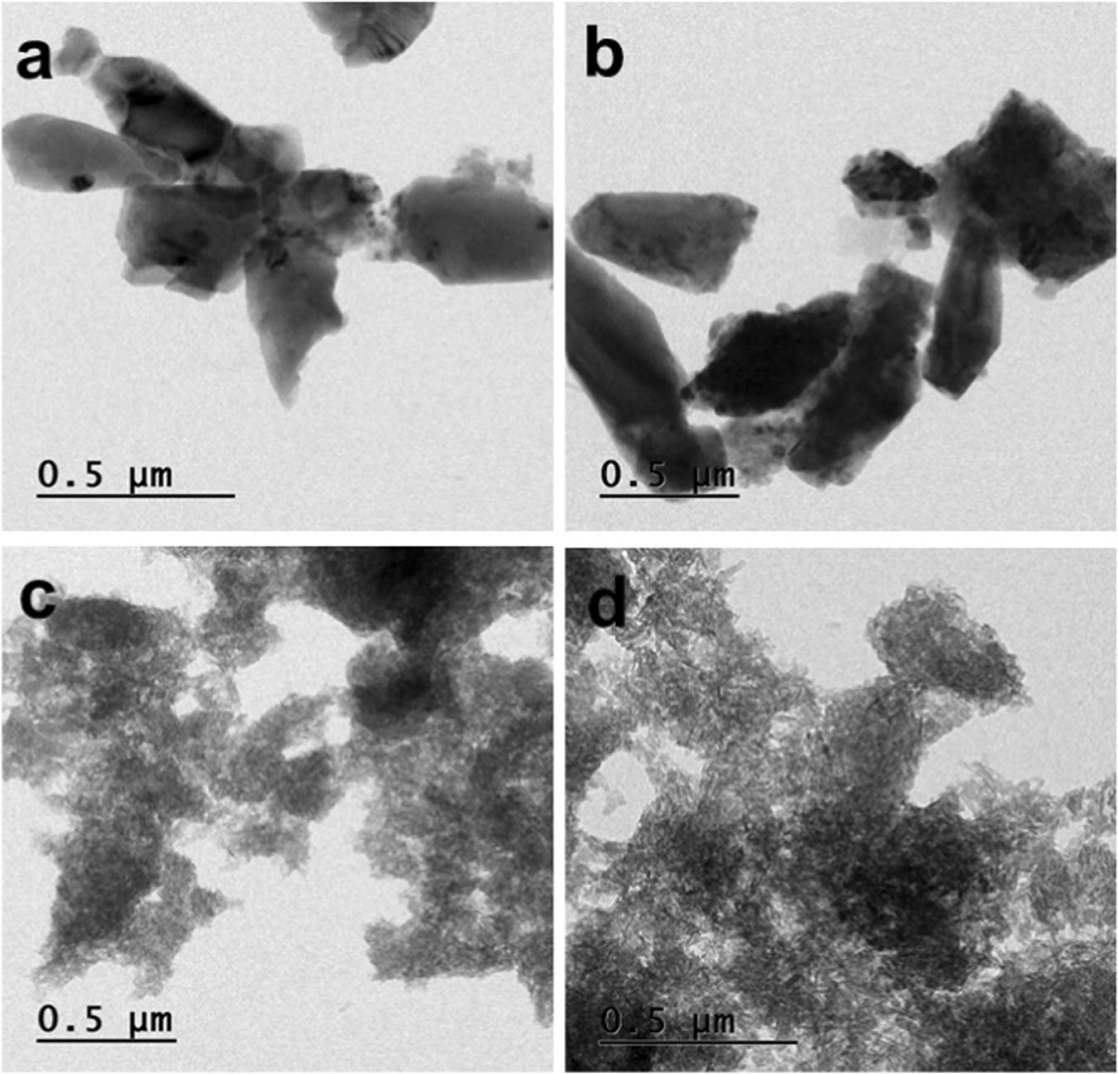
TEM morphology of **a** β-TCP, **b** β-TCP prepared in template method, **c** β-TCP/Gel-Pec composite, and **d** β-TCP/Gel-Pec/CQ composite

The prepared β-TCP by both methods and β-TCP/Gel-Pec; β-TCP/Gel-Pec/CQ composite morphology was observed through the TEM technique. Formation of some crystal type β-TCP in both methods of preparation. But β-TCP in the templated method was observed in some dense particles with good shape (Fig. 6a, b). It means the gelatin and pectin were caping the β-TCP crystal and correlated well with the SEM observations. However, β-TCP/Gel-Pec was observed in the TEM morphology in Fig. 6c. β-TCP/Gel-Pec composite was dispersed uniformly β-TCP was inside the β-TCP/Gel-Pec composite. The β-TCP is in the rod shape inside of the Gel-Pec polymers. After the CQ extract was loaded in the composite, it was noted the dense particles with uniform distribution (Fig. 6d).

### Bioactivity of composite in SBF solution

**Fig. 7:**
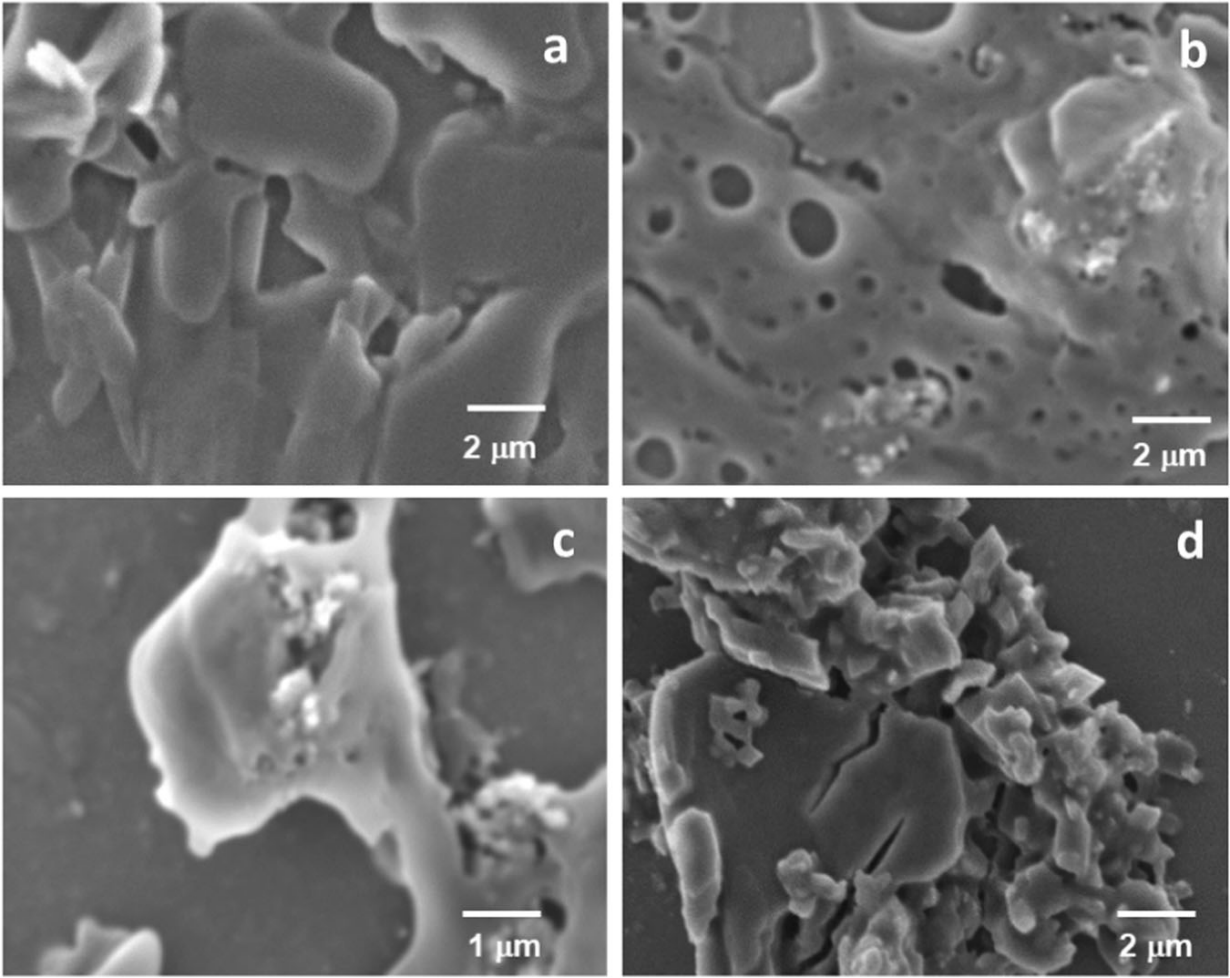
Bioactivity analysis of βtcp/Gel-Pec/CQ composite in SBF solution in 0 day (**a**), 3 days (**b**), 7 days (**c**), and 14 days (**d**)

By the templated method, the β-TCP/Gel-Pec/CQ composite was soaked for 0, 3, 7, and 14 days in SBF solution and analyzed their bone-bonding nature to find the formation of apatite crystals. The apatite formation was confirmed by morphology analysis via SEM analysis and given in Fig.7a–d. As the period upturns, from the white particle’s view, the apatite crystals are higher on the surface of the composite. On a zeroth day, the nucleation started, and for 3 days, porous was formed on the surface of the composite for HAP crystal growth. After completing 7 days of immersion, the apatite crystals were formed as the structure of a particle, and it clearly appeared at 14 days. This consequence approves the bioactive behavior of the composite, and it is biocompatible for further in vivo implantation.

### Biological studies

#### In vitro cell viability

**Fig. 8:**
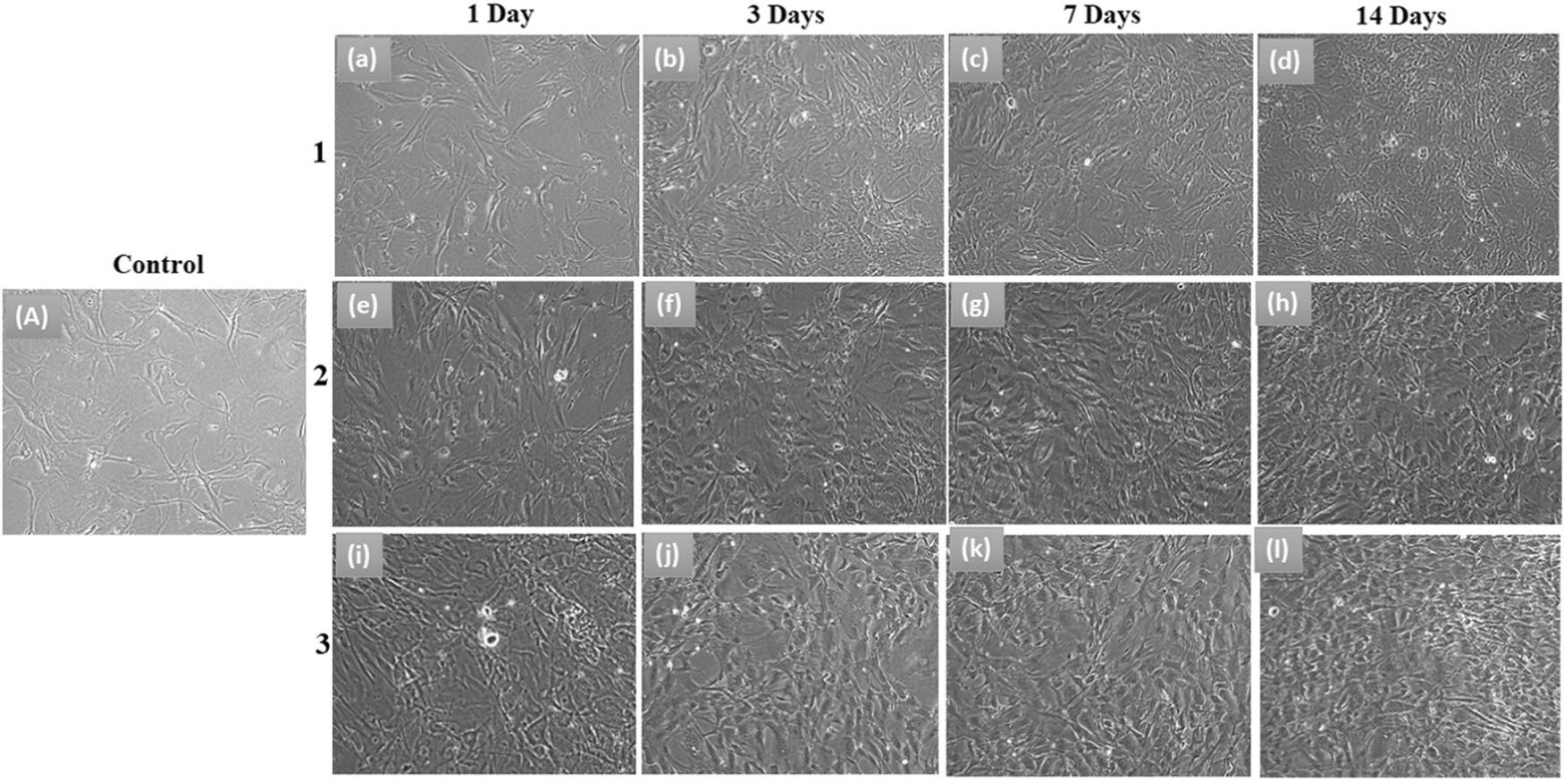
Cell proliferation assay, Optical microscopic images of hBMSCs viable on **1** β-TCP, **2** β-TCP/Gel-Pec composite, and **3** β-TCP/Gel-Pec/CQ composite for 1, 3, 7, and 14 days

Human bone marrow mesenchymal stem cells (hBMSCs) were herein taken to estimate the biocompatibility of the fabricated β-TCP, β-TCP/Gel-Pec, and β-TCP/Gel-Pec/CQ composite via the templated method preparation. Untreated cells were taken as a control in all the stages of examination. Figure 8 represents the optical microscopic images of hBMSCs after being treated with various composites for different periods. The cells appeared more compact and proliferated at 1, 3, 7, and 14 days of incubation than at control and other days of treatment. Particularly during the treatment with β-TCP/Gel-Pec/CQ composite. Besides, the counts of dead cells were minimum in this case compared with other cases. The viability of cells, as shown in Fig. 8, indicates that the composite made of β-TCP/Gel-Pec/CQ is more biocompatible and has osteogenesis potential than the other two materials as pure β-TCP and β-TCP/Gel-Pec composite. This may be due to the collective action of these β-TCP/Gel-Pec/CQ components and the addition of CQ not affecting or decreasing the biocompatible potential of β-TCP/Gel-Pec composite. Moreover, it does not produce any side effects.

**Fig. 9:**
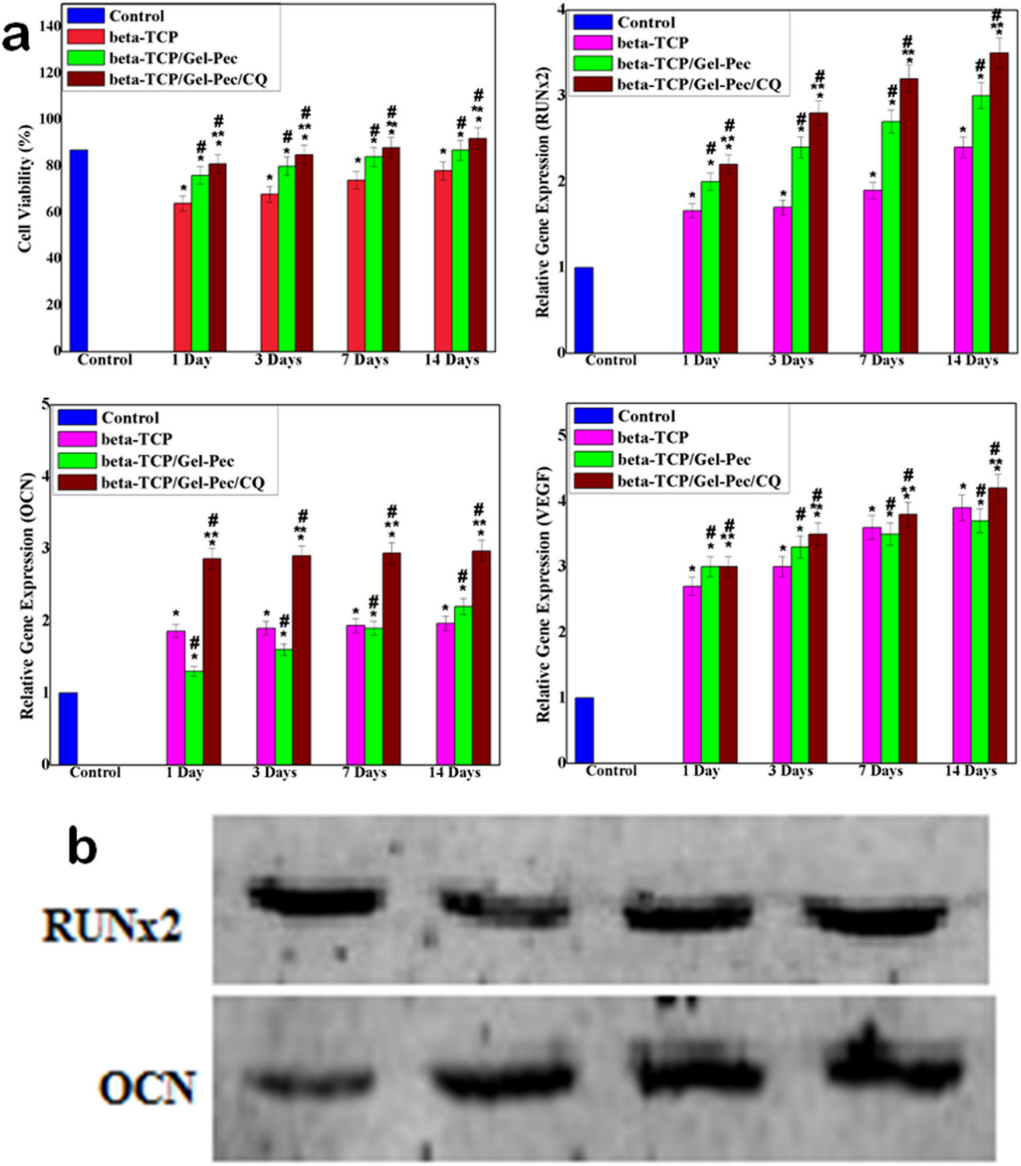
**a** (A) Quantitative MTT cell proliferation assay and RT-PCR cell differentiation analysis on the specific markers including (B) RUNx2, (C) OCN, (D) VEGF after treating with β-TCP, β-TCP/Gel-Pec, and β-TCP/Gel-Pec/CQ composites for 1, 3, 7 and 14 days of treatment. *Comparison of the indicated group with control cells within the same set. *p < 0.05. # Comparison of the indicated group with mHAP ceramic within the same set. #*p* < 0.05. **Comparison of the indicated group with β-TCP/Gel-Pec/CQ composite within the same set. **p < 0.05. **b** PCR images of RUNx2 and OCN genes

A quantitative MTT assay further confirms the above results. The highest percentage of viable cells (92%) was observed for the cells treated with β-TCP/Gel-Pec/CQ composite on the 14^th^ day of treatment. This is a significantly higher percentage than control cells and the other two composites, such as β-TCP and β-TCP/Gel-Pec. Initially, 78% of cells were viable on β-TCP ceramic on the 14^th^ day of incubation. But after the composite making with Gel-Pec polymers, the viability was greatly increased up to 87% (Fig. 9A). This result suggests the non-toxic nature of these polymers. Afterward, the antibiotic CQ extract also increased the cell proliferation rate without showing any cytotoxic effect. Finally, we can provide the conclusion that the inclusion of additional components such as polymers and antibiotic drugs on the β-TCP matrix does not induce any toxic effect on the process of osteogenic cell proliferation, and the fabricated composite β-TCP/Gel-Pec/CQ is very suitable for bone tissue regeneration applications.

#### Analysis of cell differentiation by RT-PCR analysis

RT-PCR report is used to carry out various tests like OCN, VEGF, and RUNx2 gene expression. RT-PCR (real-time Reverse transcription-polymerase chain reaction) is an important indicator for osteoblast differentiation. The RUNx2 (core-binding factor alpha 1) activities of hBMSCs on various testing samples with various culture periods such as 1, 3, 7, and 14 days are shown in Fig. 9a (B–D). All samples’ RUNx2, OCN, and VEGF activity increased as the treatment period increased. It reaches a peak value on the 14^th^ day of treatment with Sr-HAP/PSSS/PVP/LNZ composite, as shown in in vitro cell viability analysis owing to the existence of calcium phosphate and CQ extract compounds jointly. That could be clarified by the osteoconductive nature of the β-TCP/Gel-Pec/CQ composite facilitates higher RUNx2, OCN, and VEGF production. Moreover, the blot PCR analysis on the RUNx2, and OCN also shows the most increased production of osteogenesis genes as the final composite 168% and 188% for RUNx2 and OCN, respectively (Fig. 9b). The activity on the 14^th^ day was significantly higher than that of the 1^st^ day of treatment in all groups, including control groups (Untreated cells). These results suggest that along with cell proliferation, a good number of cell differentiation was also attained for hBMSCs treated with β-TCP/Gel-Pec/CQ composite.

## Discussion

Natural bioactive compounds have been applied as the main ingredient of medicines for the last few decades and have been confirmed to be enormously valuable to human health in curing and preventing diseases [40]. The traditional uses of *Cissus quadrangularis L*. include the treatment of bone fractures, scurvy, tumors, hemorrhoids, peptic ulcers, menstrual disorders, etc., and leucorrhea. The CQ is frequently used as a food supplement. The bioactive compounds, such as glycosides, polyphenols, Vitamin C, and -sitosterol, may have antiulcer qualities or speed up the healing process by releasing polyamines and TGF [41]. The biologically important rich secondary metabolites present in the CQ combined with the biomaterials were reported by researchers for the past few decades in the biomedical field. A few years back, Tamburaci et al. reported a CQ extract-loaded chitosan/Na-carboxymethyl cellulose scaffold for bone research [42]. The composite has osteoinductive properties from their investigation, and they get evidence that the scaffold can serve as a potential biomaterial for bone tissue regeneration [42]. Similarly, Layer-by-layer construction of an osteoinductive scaffold, such as an electrospun polycaprolactone scaffold, employing CQ extract and graphene oxide. Comparative studies were conducted between the modified PCL-GO-CQ scaffold and a polycaprolactone scaffold that had merely been coated with graphene oxide. Without the need for a differentiation medium, the CQ compounds present in the scaffold spontaneously encourage osteoblast development [43].

Due to contemporary demand, a high therapeutic index is now required for bone tissue engineering scaffolds. Natural therapeutic compounds that act as a modulator for new bone production are of the utmost relevance [44]. Here the phytoconstituents-filled composite was produced by incorporating CQ extract with gelatin (Gel) and pectin (Pec) polymers collective through β- tricalcium phosphate (β-TCP) bioceramic via a green template method. The effect of CQ-filled composite morphology and chemical structural properties, in vitro cytotoxicity, cell proliferation, and differentiation was investigated. The FTIR spectroscopy analysis confirmed the β-TCP/Gel-Pec/CQ composite formation. The interaction of the phosphate group in the gelatin molecules was observed as the β-TCP formation in the gelatin-templated method. It shows the gelatin bands at 3506 cm^-1^, corresponding to the presence of the primary amide bond’s NH stretching. In a similar manner, the interaction of the tricalcium phosphate/gelatin composite scaffolds was seen when they were combined with gentamycin-loaded chitosan microspheres [45]. The bioceramic composite with the polymers such as gelatin, pectin, and Calcium Phosphate (BCP) was combined to form the porous type of composite, which could absorb a rich of water molecules and mimic the extracellular matrix with the enhancement of cell proliferation, differentiation, and metabolites [46].

The ability of the composite’s bone-bonding nature will be evidence of the bioactive nature of that composite. It is a significant cause in predicting the success of in vivo implantation. The process of osseointegration will be faster from the formed apatite particles, which will help create fresh bone. The calcium phosphate ions simulate human plasma for apatite formation [47]. The progressive process of new bone development surrounding implanted materials extends from the endosteum to the material’s surface. Acidic proteins start the formation of hydroxyapatite crystals on collagen type I fibrils, which results in the mineralization of bone. Here the investigation of apatite crustal formation on the surface of the materials is evidence for the bone formation properties [24, 30]. In addition, genes like OCN, VEGF, and RUNx2 expression analysis by RT-PCR report is evidence for the biocompatible and has osteogenesis potential of the materials. Several scientists have already well known the method of forming the apatite layer on the implant’s surface [48, 49]. The calcium ions in the SBF solution were initially electrostatically connected to the –Ve charges hydroxyl substrate ion in the TCP, producing a positively charged substrate ion. Secondly, due to phosphate deposition on the cationic Ca^2+^ layer, the apatite layer was created, contributing favorably to the creation of the solid mineral surface of the bones. The β-TCP/Gel-Pec/CQ composite is the hydroxyl ion group as an extra nucleation site for biomineralization. Current research has improved the apatite layer mineralization activity by combining Gel and Pec polymers.

## Conclusion

The number of synthesized bone graft materials is prepared based on ceramic materials with different methods and materials. Here, we have developed a tricalcium phosphate-based ceramic composite combined with gelatin and pectin polymers. Phyto-estrogenic steroids *property and* carotene, ascorbic acid, and calcium contain methanolic extract of *cissus quadrangularis* (CQ) was added in the composite. The osteogenesis studies show that the β-TCP/Gel-Pec/CQ composite can express the RUNx2 and OCN genes. The prepared composite with desirable compatibility and hBMSCs proliferation ability. The evidence also confirmed their physicochemical properties with their structural and morphological confirmations. Since the β-TCP/Gel-Pec/CQ composite is potential material for bone regeneration as the lead component of tricalcium phosphate.
